# The mitochondrial unfolded protein response (UPR^mt^): shielding against toxicity to mitochondria in cancer

**DOI:** 10.1186/s13045-022-01317-0

**Published:** 2022-07-21

**Authors:** Joseph R. Inigo, Dhyan Chandra

**Affiliations:** Department of Pharmacology and Therapeutics, Roswell Park Comprehensive Cancer Center, Elm and Carlton Streets, Buffalo, NY 14263 USA

**Keywords:** Mitochondrial unfolded protein response, Mitochondrial chaperonins, Mitochondrial proteases, Mitochondrial proteostasis, Cancer

## Abstract

Mitochondria are essential for tumor growth and progression. However, the heavy demand for mitochondrial activity in cancer leads to increased production of mitochondrial reactive oxygen species (mtROS), accumulation of mutations in mitochondrial DNA, and development of mitochondrial dysfunction. If left unchecked, excessive mtROS can damage and unfold proteins in the mitochondria to an extent that becomes lethal to the tumor. Cellular systems have evolved to combat mtROS and alleviate mitochondrial stress through a quality control mechanism called the mitochondrial unfolded protein response (UPR^mt^). The UPR^mt^ system is composed of chaperones and proteases, which promote protein folding or eliminate mitochondrial proteins damaged by mtROS, respectively. UPR^mt^ is conserved and activated in cancer in response to mitochondrial stress to maintain mitochondrial integrity and support tumor growth. In this review, we discuss how mitochondria become dysfunctional in cancer and highlight the tumor-promoting functions of key components of the UPR^mt^.

## Introduction

**Fig. 1:**
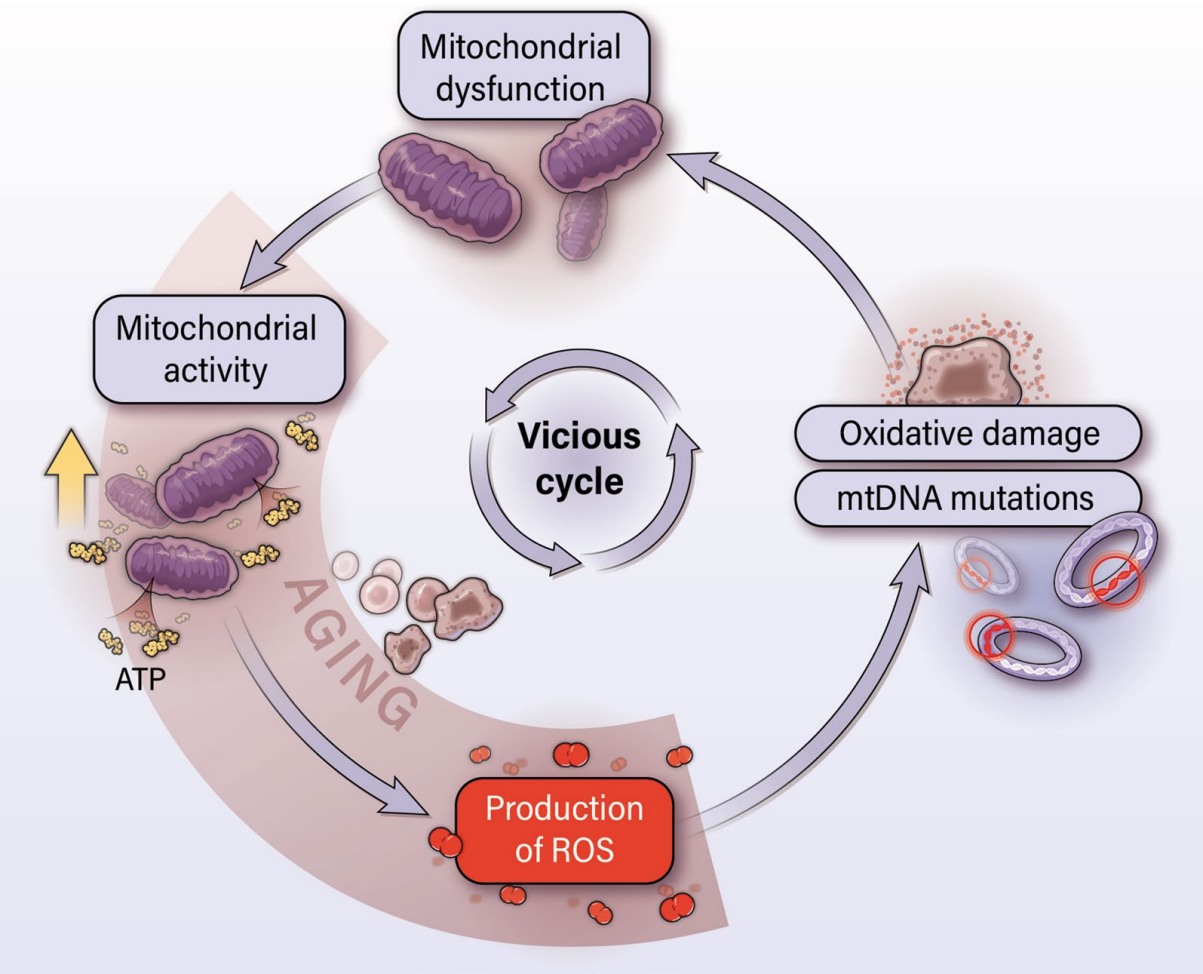
Mitochondrial dysfunction develops in aging cells and cancer cells. Increased mitochondrial activity in cancer cells leads to an increased production of mtROS. Over time, mtROS damage mtDNA and cause the accumulation of mutations in mtDNA. This leads to further increases in mtROS, which eventually give way to mitochondrial dysfunction. Thereafter, a vicious cycle occurs in which mitochondrial dysfunction increases mtROS and leads to a deeper state of mitochondrial dysfunction. Cancer cells can utilize dysfunctional mitochondria to promote tumor growth and progression

As cancer cells undergo uncontrolled proliferation, these cells develop a number of vulnerabilities that require protection by key support systems [1, 2]. Mitochondria, in particular, contribute to growth and survival throughout the various stages of cancer [3]. However, mitochondria also undergo genetic alterations resulting in a dysfunctional electron transport chain, which generate excessive levels of mitochondrial reactive oxygen species (mtROS) (Fig. 1) [4–6]. Under physiological conditions and during the early stages of disease, mitochondria produce moderate levels of mtROS that are beneficial to cellular growth and survival. However, as mitochondrial dysfunction worsens, levels of mtROS can exceed the tolerable threshold and become lethal to tumor cells [7–9]. The mtROS promote unfolding and aggregation of mitochondrial proteins, leaving mitochondria in an increasingly fragile and dysfunctional state [4–6].

The mitochondrial unfolded protein response (UPR^mt^), a mitochondrial stress response observed in *C. elegans* and mammalian system [10, 11], serves as an important support system in cancer by maintaining mitochondrial integrity and promoting tumor growth [12, 13]. UPR^mt^ activates a series of chaperones and proteases that alleviate the damaging effects of mtROS. Increasing evidence suggests that the UPR^mt^ is conserved between *C. elegans* and mammals [14].

**Fig. 2:**
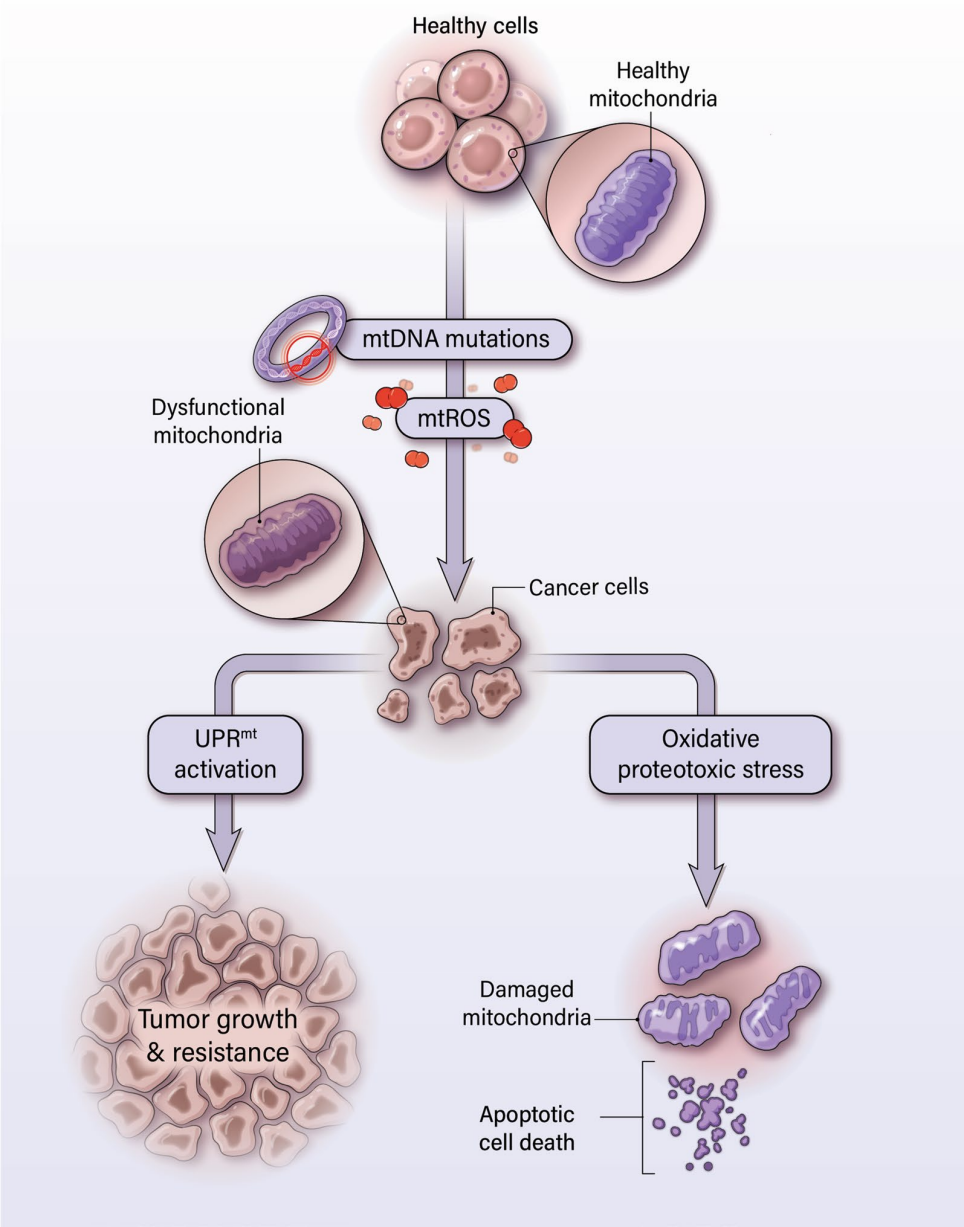
Mitochondrial unfolded protein response (UPR^mt^) is proposed to mediate mitochondrial maintenance in cancer. Cancer cells develop mitochondrial dysfunction due to increased accumulation of mtDNA and mtROS relative to non-malignant cells. Although mtROS can be beneficial to cell growth, excess mtROS can damage proteins and further propagate oxidative and proteotoxic stress in the mitochondria. The UPR^mt^ system refolds proteins so that they return to their proper conformation or cleaves such proteins. This preserves mitochondrial integrity and prevents mitochondrial apoptosis

The current review elaborates the development of mitochondrial dysfunction in cancer and discusses how mitochondrial dysfunction and elevated mtROS benefit tumor growth. We discuss the ability of UPR^mt^ to prevent functional decline of mitochondria in supporting tumor growth and progression (Figs. 1 and 2). In examining how UPR^mt^ preserves mitochondrial health, we discuss the individual functions of UPR^mt^ components, their associations with clinical outcomes, and their tumor-promoting roles.

## Non-oncogene addiction

Targeting of oncogenes has always been an enticing approach in cancer therapy [15]. Oncogenes such as rat sarcoma virus (*RAS)* [16], myelocytomatosis (*MYC)* [17], and epidermal growth factor receptor (*EGFR)* [18] serve as key drivers of tumor initiation and growth [19, 20]. However, we often overlook the importance of support systems that maintain the tumorigenic state. As cancer cells undergo rapid proliferation, cancer cells bear genetic alterations that produce multiple stress phenotypes including DNA damage stress, mitotic stress, metabolic stress, oxidative stress, and proteotoxic stress [1, 2]. These vulnerabilities, if left unchecked, can be lethal to tumor viability. In order to overcome these challenges, stress response pathways are recruited to alleviate stress, allow cell survival, and promote tumor progression. The genes associated with stress response may not necessarily have the classical features of oncogenes, such as activating mutations or overexpression that can directly induce carcinogenesis, and are therefore referred to as non-oncogenes [21]. Nonetheless, these non-oncogenes are fundamental to tumor maintenance and the increased reliance of cancer cells upon these non-oncogenes is referred to as “non-oncogene addiction” [21].

The current review focuses on the vulnerability of mitochondria in cancer cells. Extensive ROS production by mitochondria along with other events such as alterations in the antioxidant system leads to oxidative stress in cancer cells [22]. Reactive oxygen species (ROS) then induce the unfolding/misfolding and aggregation of proteins within the mitochondrion to propagate proteotoxic stress [23, 24]. Genes of a mitochondria-specific stress response, known as the UPR^mt^, relieve the constant oxidative and proteotoxic stress present in mitochondria. In doing so, activation of UPR^mt^ functions as a form of non-oncogene addiction in cancer. The following section describes how mitochondria become dysfunctional in cancer, yet continue to contribute to cancer progression, and highlights the specific vulnerabilities that pressure mitochondria to increasingly rely upon UPR^mt^.

## Mitochondrial dysfunction and cancer

Mitochondria are key organelles critical for fulfilling the bioenergetic and biosynthetic needs of the cellular system [25]. Mitochondria contain their own genome, which encodes for 13 proteins, 22 tRNAs, and 2 rRNAs [26]. All 13 mitochondrial gene-encoded proteins are part of the oxidative phosphorylation (OXPHOS) Complexes in the electron transport chain (ETC) that facilitate ATP production [27]. Mitochondria are highly dynamic networks that perform fusion and fission to: allow communication and distribution of resources between mitochondrial compartments, preserve genomic integrity, and maintain mitochondrial homeostasis [28, 29]. The mitochondrial metabolism also generates oncometabolites such as (R)-2-hydroxyglutarate (2-HG) via isocitrate dehydrogenase (IDH) mutation in acute myeloid leukemia (AML), succinate via mutation succinate dehydrogenase (SDH), and fumarate via mutation in fumarate hydratase (FH), which promote tumor growth and progression [3, 30, 31]. The following sections will elaborate on how the accumulation of mutations in mitochondrial DNA (mtDNA) and increases in mitochondrial reactive oxygen species (mtROS) contribute to the development of mitochondrial dysfunction in cancer [23], and how mitochondrial dysfunction propagates the growth and survival of cancer cells (Figs. 1 and 2).

### Mitochondrial ROS production and mitochondrial mutagenesis

Mitochondria are a major source of ROS, due to the activity of protein complexes of the ETC [4, 32]. Within the ETC, the transfer of electrons between complexes is coupled with the movement of protons across the inner mitochondrial membrane to generate an electrochemical proton gradient that drives ATP synthesis [33]. During this process, electrons can leak from the ETC complexes and interact with oxygen to form mtROS such as superoxide, hydrogen peroxide, and hydroxyl radicals [4, 5]. In addition, enzymes of the tricarboxylic acid (TCA) cycle located in the mitochondrial matrix also contribute to mtROS production. For example, 2-oxoglutarate dehydrogenase, branched-chain 2-oxoacid dehydrogenase, pyruvate dehydrogenase complexes, glycerol-3-phosphate dehydrogenase (mGPDH), electron-transferring flavoprotein–ubiquinone oxidoreductase (ETF-QOR), dihydroorotate dehydrogenase (DHODH), and p66shc/cytochrome c system produce superoxide and hydrogen peroxide [34, 35]. These highly reactive species can promote oxidative damage to surrounding proteins, lipids, and DNA [36], but are rapidly quenched by antioxidant enzymes under most circumstances [37].

As an organism ages, mtDNA suffers extensive oxidative damage due to the proximity of mtDNA to mtROS production [38]. The lack of protection by histones and deficiency in DNA repair mechanisms relative to nuclear DNA further lead to the vulnerability of mtDNA [39]. As a result, mtDNA undergoes a greater rate of mutations compared to nuclear DNA. The accumulation of mutations, including point mutations, insertions, deletions, and alterations in mtDNA copy number [40], further exacerbates mtROS generation, leading to a self-enforcing cycle of mtDNA damage and mtROS production [41]. These ongoing activities ultimately result in a state of mitochondrial dysfunction in which various functions of the mitochondria, including electron transfer by the ETC and ATP production, are impaired during the aging process [6, 42]. Cancer cells develop mitochondrial dysfunction in a similar fashion (Fig. 1) [6]. The increased mitochondrial activity required by cancer cells leads to increased production of mtROS. This results in elevated levels of oxidative damage and mtDNA mutations, leading to mitochondrial dysfunction. A vicious cycle develops in which mitochondrial dysfunction aggravates mtROS generation, leading to further mitochondrial dysfunction (Fig. 1). However, as we will discuss in the following sections, cancer cells use the mitochondrial dysfunction to their advantage throughout the progression of cancer [43].

### The role of mitochondria in tumorigenesis

Although the link between mitochondrial dysfunction and tumorigenesis is not fully clear, some evidence points to the role of mitochondrial dysfunction and oxidative stress in malignant transformation. Severe mutations in the tRNALys gene of mtDNA (m.8363G > A) that critically disrupt the ETC do not support tumorigenesis, but mutations in the MT-ND1 (m.3460G > A), MT-ND4 (m.11778G > A) and MT-ND6 (m.14484 T > C) of mtDNA that mildly impair the ETC promote mild mitochondrial dysfunction and support tumorigenesis [44]. Mutations in the ATP synthase subunit 6 gene (*ATP6*) in mtDNA can increase superoxide production from Complexes I, II, and III to provide an advantage in early tumor growth [45]. The T8993G mutation in *ATP6* specifically favors tumorigenicity in prostate cancer (PCa) [46]. A heteroplasmic mutation in the NADH dehydrogenase subunit 5 gene (*ND5*), identified in colorectal tumors [47], disrupts the synthesis of the ND5 subunit of Complex I and subsequently hinders the proper assembly of Complex I. This causes an increase in mtROS levels and enhanced tumorigenicity [48]. Furthermore, increases in mtROS due to the Kirsten rat sarcoma virus (KRAS) drive the formation of pancreatic precancerous lesions [49]. Overall, key features of mitochondrial dysfunction, mutations in mtDNA and increases in mtROS, appear to support tumor development as early as the tumor initiation stage.

### Mitochondrial ROS and cellular signaling

Mitochondrial ROS show beneficial effects in cancer cells via direct induction of multiple signaling events for cell growth. For example, hydrogen peroxide can inactivate the tumor suppressor phosphatase and tensin homolog (PTEN) by oxidizing its essential cysteine residue in various types of cells [50, 51]. This leads to the accumulation of phosphatidylinositol 3,4,5-trisphosphate (PIP3) followed by signaling through the Ak strain transforming (AKT) pathway that promotes the growth and survival of cancer cells [50, 52, 53]. Hydrogen peroxide also drives tumorigenesis via the AMP-activated protein kinase (AMPK) pathway [54] and can inactivate Cdk1-opposing phosphatases to allow cyclin-dependent kinase 1 (Cdk1)-mediated mitotic progression [55]. Production of mtROS from Complex III of the ETC is essential for KRAS-mediated mitogen-activated protein kinase (MAPK)/extracellular signal-regulated kinase (ERK) signaling for cell proliferation and tumorigenicity [56]. Additionally, mtROS can trigger protein kinase D1 (PKD1), thereby activating transcription factors nuclear factor kappa B1 (NF-κB1) and NF-κB2 to upregulate EGFR signaling [49]. These direct roles of mtROS in activating proliferative pathways highlight the tumor-promoting role of increased mtROS production by dysfunctional mitochondria.

### Metabolite formation and tumor growth

Mitochondrial dysfunction increases levels of high-mobility group box 1 (HMGB1) [57]. HMGB1 is upregulated in cancer and is released into the extracellular space to allow interaction with the receptor for advanced glycation end products (RAGE) [58]. This induces a signaling pathway in which RAGE phosphorylates Complex I to enhance ATP production [59]. Moderate mitochondrial defects due to mtDNA mutations promote integration of glutamine into the TCA cycle by conversion of glutamine to glutamate, thereby fueling the TCA cycle for the production of ATP [60]. During inflammation-associated pancreatic tumor development, an increase in mitochondrial fatty acid *β*-oxidation occurs [61]. Elevated fatty acid *β*-oxidation activity has been observed in tumor spheres and is associated with increased NADH and FADH_2_ [62]. Therefore, increased fatty acid *β*-oxidation-mediated generation of ROS and altered energy metabolism in mitochondria promote tumor growth and progression. Overall, dysfunctional mitochondria can increase the production of ATP-generating metabolites to meet the increased metabolic demands of tumor growth.

### Tumor microenvironment and inflammation

Tumor cells in the tumor microenvironment (TME) recruit non-malignant cells to promote tumor growth and further propagate mitochondrial dysfunction [63, 64]. Among the non-malignant cells of the TME, inflammatory immune cells contribute to mitochondrial dysfunction by directly releasing ROS into the TME [65, 66] or secreting pro-inflammatory cytokines [67]. For example, macrophages and neutrophils can release various forms of ROS to damage the DNA of neighboring cells [68]. Likewise, immune cells can secrete factors such as tumor necrosis factor alpha (TNF-*α*), interleukin 1 beta (IL-1*β*), and interferon-gamma (IFN-*γ*) to alter mitochondrial membrane potential, inhibit the ETC, increase proton leak, and ultimately stimulate the production of mtROS [69–73]. This underscores the idea that mitochondrial dysfunction does not always occur due to the activity of tumor cells alone, but may involve non-tumor cells within the TME.

### Adaptation to hypoxia

As tumors grow, the demand for oxygen and nutrition exceeds the availability of sufficient vasculature for supporting tumor proliferation. This leads to a state of hypoxia in various malignancies [74]. In response, hypoxia-inducible factor-1*α* (HIF-1*α*) signaling is activated to continue the growth and survival of the tumor [75]. Normally, HIF-1*α* is destabilized by prolyl hydroxylases (PHDs) in the presence of oxygen [76]. However, as oxygen levels decline during hypoxia, PHD activity is inhibited and HIF-1*α* is stabilized [74]. During hypoxic conditions, mitochondria generate excess mtROS from Complex III of the ETC, promoting the stabilization of HIF-1*α*. [77, 78]. Even respiration incompetent cancer cells, due to defects in Complex III, can still produce increased mtROS to stabilize HIF-1*α* during hypoxia [79]. Altogether, this indicates that dysfunctional mitochondria, despite impairments in ETC, assist in HIF-1*α* stabilization through the production of mtROS.

### Mitochondrial DNA mutations and resistance to cell death

Mitochondrial dysfunction also contributes to deficits in the cell death pathways. Various mutations in mtDNA modulate the apoptotic response. For example, partial deletions in mtDNA offer protection against mitochondria-initiated cell death [80]. The previously mentioned mutations in *ATP6* and *ND5* raise the apoptotic threshold of tumor-forming cells, allowing resistance against oxidative stress-induced cell death [45, 48]. In vivo, a combination of mtDNA mutations in *ND4*, *ATP6*, and *16S rRNA* genes or in the *COI* and *ND3* genes confers resistance to 5-fluorouracil- and cisplatin-induced apoptosis [81]. In ovarian cancer, mutations in *ND4* lead to acquired chemoresistance against paclitaxel and carboplatin [82]. Gefitinib-mediated mitochondrial dysfunction in non-small cell lung cancer (NSCLC) cells is proposed to aid in the development of drug resistance [83, 84]. The G10398A substitution in *ND3* produces apoptotic resistance to etoposide [85]. Although counterintuitive, numerous mutations in the mitochondrial genome actually enhance the survival of cancer cells [86, 87].

### Mitochondrial DNA mutations, mtROS, and metastasis

Metastasis is a process observed in late-stage disease in which tumor cells disseminate to secondary sites. Metastasis is the leading cause of mortality in patients [88, 89]. Mutations in mtDNA, particularly in the *ND* genes, enhance the metastatic activity of cancer cells. In breast cancer, the G10398A substitution in *ND3* increases the number of metastatic foci in the lungs of mice [85]. Similarly, missense mutations in breast cancer such as C12084T in the *ND4* gene and A13966G in the *ND5* gene are associated with defects in mitochondrial respiration and augmented metastatic potential independent of ROS overproduction [90]. Highly metastatic breast cancer cells exhibit downregulation of mitochondrial transmembrane protein 126A (TMEM126A), which results in mitochondrial dysfunction, as shown by altered mitochondrial membrane potential and increased mtROS [91]. This decrease in TMEM126A has been shown to promote epithelial–mesenchymal transition (EMT), extracellular matrix (ECM) remodeling, cell adhesion, and increased lung metastasis [91]. In lung cancer, the G13289A mutation in *ND5* increases invasive activity [92]. G13997A and 13885insC mutations in *ND6* produce defects in Complex I of the ETC, leading to an overproduction of mtROS and an increase in the metastatic potential of lung carcinoma cells [93]. Likewise, *ND6* nonsense and missense mutations are associated with higher rates of lymph node metastases in human lung cancer by promoting migratory and invasive activities [94]. The 13885insC mutation in *ND6* is associated with the overexpression of metastasis-related genes and promotes metastasis in various cancers [95]. In addition, a study of cancer stem cells demonstrates that a subset of these cells maintain elevated mtROS, which serve to activate MAPK and promote EMT in order to increase metastatic potential [62]. Even as tumors approach late-stage disease, dysfunctional mitochondria are able to assist in tumor development at secondary sites.

Overall, mitochondria contribute to various stages of cancer development and progression despite their dysfunctional state [3]. Moreover, cancer cells are able to take advantage of the mtDNA mutations and elevated mtROS levels associated with mitochondrial dysfunction to promote growth and survival. However, as cancer cells continue to propagate in a highly proliferative manner, mitochondrial dysfunction can worsen and become lethal (Figs. 1 and 2) [6]. The resulting increases in oxidative stress due to mtROS lead to further proteotoxic stress, which is marked by oxidative damage, protein unfolding, and protein aggregation [36, 96]. This leaves mitochondria in a fragile state and pushes cells to the brink of cell death. To adjust to elevated mtROS, cancer cells maintain oxidative status at a level that benefits, but does not become toxic to cancer cells [7–9].

## Relieving mitochondrial stress through antioxidants

Mitochondria rely upon antioxidants to quench mtROS. Antioxidant enzymes such as superoxide dismutase (SOD), catalase, and glutathione peroxidase scavenge excess mtROS [37]. SODs allow the dismutation of superoxide radicals into hydrogen peroxide and oxygen. Catalases and peroxidases further process hydrogen peroxide into water [37]. An enhanced antioxidant response is commonly observed in cancer [97]. However, levels of individual antioxidants can vary and are, at times, lower in tumors compared to normal tissue [98–103]. As mtROS levels exceed the capacity of the antioxidant system due to mitochondrial dysfunction, another mechanism must be at play in order to preserve mitochondrial health.

## Relieving mitochondrial stress through the mitochondrial unfolded protein response (UPR^mt^)

As mentioned, chronic oxidative stress due to inherent mitochondrial dysfunction leads to proteotoxic stress: unfolding, misfolding, and aggregation of mitochondrial proteins. If left unresolved, the accumulation of protein aggregates can further increase oxidative stress, leading to a feedback loop that propagates mitochondrial dysfunction [104]. For example, disruption of proteins in the ETC enhances the leakage of electrons and leads to increased mtROS [105]. The following section will discuss a mitochondrial stress response, consisting of chaperones and proteases, which halts this harmful feedback loop by directly targeting proteotoxicity in mitochondria (Fig. 2).

### Early signs of a mitochondrial stress response

**Fig. 3:**
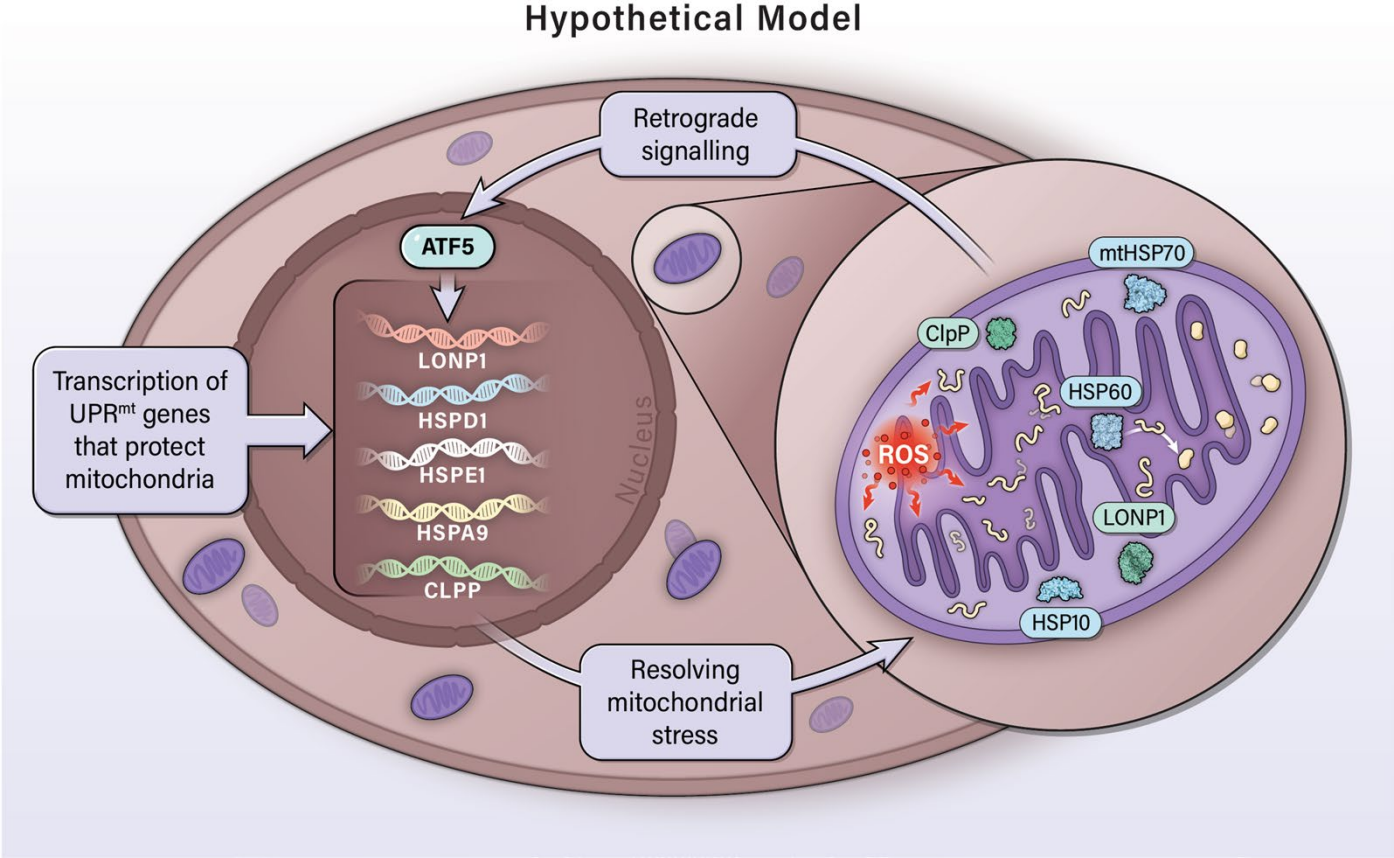
Hypothetical model of the mitochondrial unfolded protein response (UPR^mt^) signaling in cancer. Mitochondrial dysfunction increases mtROS (oxidative stress), which damages proteins in the mitochondria and cause the unfolding and aggregation of mitochondrial proteins (proteotoxic stress). In response, the transcription factor ATF5 induces the upregulation of mitochondrial components to ease proteotoxic stress. Chaperones HSP60, HSP10, and mtHSP70 mediate the refolding of proteins into their proper conformation. Proteases LONP1 and ClpP cleave and dispose of any additional damaged proteins that did not undergo processing by HSPs. Together, this system maintains mitochondrial integrity in the face of continuous oxidative and proteotoxic stress in cancer

Signs of a mitochondrial stress response involving chaperones and proteases emerged as early as 1996, when Martinus et al. discovered that generating mitochondria-specific stress, by depletion of mtDNA, induces the transcriptional activation of mitochondrial chaperones’ heat shock protein 60 (HSP60) and heat shock protein 10 (HSP10) in rat hepatoma cells [11]. Further evidence of this mitochondrial stress response was shown by a study in which the accumulation of protein aggregates in the mitochondrial matrix induced the transcriptional upregulation of mitochondria-specific genes that code for chaperones HSP60 and HSP10, as well as the protease, caseinolytic protease (ClpP) [106]. Altogether, these chaperones and proteases were shown to reduce mitochondrial protein aggregation (Fig. 3) [106].

Various proteins involved in the mitochondrial stress response are encoded by the nuclear DNA [11, 106] and must be imported into the mitochondria [107, 108]. Therefore, mitochondria-to-nucleus communication during mitochondrial stress plays a critical role in maintaining mitochondrial homeostasis or functions. This process is known as mitochondrial retrograde signaling, in which mitochondria send a signal to the nucleus in order to regulate nuclear gene expression and allow mitochondrial homeostasis (Fig. 3) [109, 110].

### *C. elegans * as a model to study UPR^*mt*^

The *C. elegans* model has been integral in understanding this mitochondria-specific retrograde stress response, now known as the UPR^mt^. During UPR^mt^, defective protein folding in mitochondria transactivates *hsp-60* and *hsp-6*, which encode chaperones HSP60 and mitochondrial HSP70 (mtHSP70), respectively [111], as well as *clpp-1*, which encodes ClpP. [112]. Using *C. elegans*, Nargund et al. demonstrated that the transcription factor ATFS-1 is key to activating the UPR^mt^ response [113]. ATFS-1 contains both a nuclear localization sequence (NLS) and mitochondrial targeting sequence (MTS). Under non-stress conditions, ATFS-1 is imported into the mitochondria and is rapidly degraded by the protease, Lon peptidase 1 (LONP1). However, during mitochondrial stress, proteotoxicity impairs protein import into the mitochondria and, consequently, ATFS-1 accumulates in the nuclei to induce the expression of a broad variety of genes involved in mitochondrial stress, including *hsp-60* [113].

Another study by Nargund et al. used ChIP-Seq to identify 381 genes induced by ATFS-1 during mitochondrial stress [114]. Of these genes, ATFS-1 interacts with 70 of their corresponding promoters. Notably, ATFS-1 binds and suppresses mtDNA-encoded OXPHOS genes in both the nuclear and mitochondrial genomes. Concurrently, ATFS-1 binds promoters of NADH ubiquinone oxidoreductase assembly factors to aid in ETC complex. This indicates that ATFS-1 limits further transcription of OXPHOS genes while maintaining OXPHOS complexes already present, in order to optimize respiratory activity during mitochondrial stress [114]. Protein synthesis in the cytoplasm is also reduced during UPR^mt^ [115], presumably to reduce cell stress. Speculations remained as to whether mammalian cells similarly utilize a regulated UPR^mt^ system. The individual works of Nargund, Wu, Schulz, and Fiorese et al. began to fill this gap in knowledge [10, 14, 113, 116].

### *Transcription factor ATF5 and the mammalian model of UPR*^*mt*^

Transcriptomic and proteomic analysis later revealed strong conservation in the UPR^mt^ response between *C. elegans*, mice, and humans. Genes encoding chaperones (*Hspd1*, *Hspe1*, *Hspa9*) and proteases (*Clpp*, *Lonp1*) in *C. elegans* also associate with UPR^mt^ homologs in mammals [14]. Over 400 genes were found to be activated in the mammalian UPR^mt^ response [14, 113], which can be categorized by various functions including mitochondrial biogenesis, metabolism, protein folding quality control, and ROS detoxification [116]. Recently, Fiorese et al. identified activating transcription factor 5 (ATF5) as the mammalian homolog of ATFS-1 [10]. When ATF5 was expressed in worms lacking ATFS-1, it induced *hsp-60* during mitochondrial stress but not during endoplasmic reticulum (ER) stress, indicating organelle specificity. In HEK293T cells, elevated mtROS activated ATF5-mediated transcription of the genes for HSP60, mtHSP70, and LONP1. Again, ER chaperones were not induced by ATF5 under these conditions. Similar to ATFS-1, ATF5 contains both an NLS and MTS, allowing ATF5 to shuttle between the mitochondria and nucleus during UPR^mt^. Knockdown of ATF5 in HEK293T reduced proliferation, mitochondrial respiration, and induction of HSP60, mtHSP70, and LONP1. Overall, this study highlights ATF5 as the transcription factor that specifically mediates mammalian UPR^mt^ and is independent of the ER stress response [10].

### *Other transcription factors in UPR*^*mt*^

In addition to ATF5, other transcription factors have been implicated in the UPR^mt^. Transcription factor ATF4 induces the expression of another transcription factor CCAAT/enhancer-binding protein (C/EBP) homologous protein (CHOP) [117, 118]. CHOP, in turn, dimerizes with transcription factor C/EBP*β* to function as a regulator of the mitochondrial stress response [106]. In breast cancer, misfolded proteins in the mitochondria activate estrogen receptor alpha (ER*α*), which increases the transcription of the intermembrane space protease OMI and increases the activities of the proteasome [119]. ER*α*-negative breast cancer cells respond to mitochondrial stress by increasing the expression of sirtuin deacetylase 3 (SIRT3), leading to the deacetylation of the transcription factor FOXO3a [120]. Forkhead box protein O3a (FOXO3a) then translocates to the nucleus to promote the transcription of antioxidants superoxide dismutase 2 (SOD2) and catalase [120]. Sirtuin deacetylase 7 (SIRT7) can alleviate protein folding stress in hematopoietic stem cells, partly by inducing the expression of canonical UPR^mt^ components including HSP60, HSP10, and ClpP [121]. Another transcription factor, heat shock factor 1 (HSF1), has been shown to induce the expression of UPR^mt^ chaperones in mammalian cells in response to mitochondria-specific stress [122].

Altogether, these other arms of UPR^mt^ act in tandem to maintain the integrity of the mitochondria. However, the transcription factors ATF4, CHOP, C/EBP*β*, ER*α*, and HSF1 are also activated during ER stress to promote endoplasmic reticulum unfolded protein response (UPR^er^) [117, 118, 123–129]. While overlap in the functions of ATF5 between UPR^mt^ and UPR^er^ is expected due to the known regulation of ATF5 by CHOP [130] and ATF5 having been shown to increase in expression due to ER stress [131, 132], studies continue to demonstrate the inability of ATF5 to induce chaperones during ER stress [10, 133]. Therefore, ATF5 remains to be the only mitochondria-specific transcription factor during mitochondrial stress and is viewed as the main regulator of UPR^mt^ (Fig. 3).

Mitochondrial dysfunction is known to induce *ATF5* transcripts [10, 134, 135] and transactivate UPR^mt^ targets downstream of ATF5, due to the various stressors associated with mitochondrial dysfunction: mtDNA depletion [11, 111], accumulation of unfolded proteins in the mitochondria [106, 119], inhibition of ETC activity [10, 113, 136], and deficient expression of ETC components [111]. ATF5 has been evidenced to transactivate HSP60, mtHSP70, and LONP1 [10] and likely induces HSP10 and ClpP [137] during activation of mammalian UPR^mt^. Mitochondrial chaperones HSP60, mtHSP70, and HSP10 assist in protein folding and are particularly essential to reducing the aggregation of unfolded proteins during stress conditions [138]. Mitochondrial proteases LONP1 and ClpP degrade damaged proteins during this response [139, 140]. Digested peptides can then be transported by HAF-1, a mitochondrial inner-membrane-localized ABC transporter, from the mitochondrial matrix to the intermembrane space where they are believed to diffuse into the cytoplasm [141]. Between the mitochondrial chaperone and protease systems, it is believed that cells prefer to utilize chaperones before resorting to proteases in order to manage unfolded proteins in the mitochondria [142]. In light of this, we believe that mitochondrial dysfunction in cancer produces a state of chronic mitochondrial stress, which then constitutively activates ATF5 and upregulates the expression of the UPR^mt^ components (Fig. 3).

## Roles of the UPR^mt^ components in cancer

UPR^mt^ signaling plays a critical role in cancer [143] and requires further investigation to clearly indicate its impact on tumor growth and progression. While many studies, primarily utilizing *C. elegans* and sometimes mammalian kidney cells, have confirmed the presence and function of UPR^mt^ during mitochondrial stress, the study of the UPR^mt^ as a whole system has been lacking in cancer. However, individual components of UPR^mt^ have displayed roles in tumor growth and survival. This is unsurprising due to the prevalence of mitochondrial dysfunction and oxidative stress throughout cancer development and progression. The following sections will discuss the known cancer-specific roles of ATF5 and key UPR^mt^ proteins downstream of ATF5: HSP60, HSP10, mtHSP70, LONP1, and ClpP.

### Activating transcription factor 5 (ATF5)

ATF5, part of the bZip family of transcription factors, contains a leucine zipper that allows dimerization with ATF5 and other transcription factors, and contains a basic N-terminal portion involved in DNA binding [144–146]. The structure also includes an MTS, nuclear export sequence (NES), and NLS. This allows ATF5 to translocate between different cell compartments [10]. ATF5 must homodimerize or form heterodimers with other transcription factors in order to function [146]. Binding partners of ATF5 include other ATF5 proteins, as well as C/EBP*β* [147, 148].

ATF5 is upregulated in glioblastoma [149] as well as cancers of the breast [150], pancreas [151], rectum [152], and ovaries [153]. High ATF5 expression correlates with reduced survival in glioma [154] and lung cancer patients [155]. ATF5 expression can be induced by various forms of stress: heat shock [156], amino acid depletion [157], increases in ROS [10, 131, 158], inhibition of the proteasome [131], endoplasmic reticulum stress [131, 132], and radiation [155].

As a transcription factor, ATF5 regulates the expression of various anti-apoptotic genes in cancer. For example, ATF5 regulates Egr-1 expression in glioma and breast cancer cells to mediate proliferation and survival [159]. ATF5 transactivates BCL-2 in glioma and breast cancer cells to promote survival [160]. In glioblastoma, non-small cell lung cancer, and pancreatic cancer, ATF5 regulates the protein expression of deubiquitinase USP9X, which, in turn, stabilizes B cell lymphoma 2 (BCL-2) and myeloid leukemia 1 (MCL1) [161]. In addition, ATF5 regulates B cell lymphoma-extra large (Bcl-xL) expression in glioblastoma cells [161]. Although highly probable, studies have not yet shown the ability of ATF5 to regulate the expression of UPR^mt^ components in cancer. In the following sections, the pro-tumor functions of downstream targets of ATF5 will be examined.

### Heat shock protein 60 (HSP60)

Monomers of HSP60 arrange into stacked heptameric rings that form a complex with HSP10 and ATP to create a classical theorized “football-shaped” structure that can envelop misfolded or unfolded proteins and assemble them into their proper conformation [162–164]. This is particularly essential for mitochondrial proteins, as most of these proteins are produced outside of the mitochondria and must be imported into the mitochondria in an unfolded state [107, 108]. Interestingly, during the chaperonin reaction cycle active single- (half-football) and double-ring (football) complexes coexist for the proper folding of proteins [164]. The HSP60–HSP10 complex is crucial during high oxidative stress, as ROS can also directly oxidize mitochondrial proteins to promote protein aggregation [104, 165]. Indeed, protein aggregation has been shown to cause the accumulation of HSP60 in the mitochondria [166]. In addition, HSP60 can play a role in posttranslational modifications [167].

HSP60 is overexpressed in various cancers. Specifically, HSP60 is upregulated early in prostate carcinogenesis [168] and large bowel carcinoma [169], increases during the progression of hepatocarcinogenesis [170], and indicates a greater risk for progression of urothelial tumors of the bladder [171]. High HSP60 levels correlate with advanced tumor grade in PCa [172], pancreatic cancer [173], and large bowel carcinoma [174]. HSP60 is increased in the serum of patients with metastatic colorectal cancer [175], is associated with the presence of lymph node metastases in large bowel carcinoma [174], and correlates with deep invasion and lymph node metastasis in gastric cancer [176]. Furthermore, elevated HSP60 levels indicate reduced survival in patients with PCa [172], gastric cancer [176], and neuroblastoma [177].

Knockdown of HSP60 decreases cell proliferation in pancreatic cancer [173], ovarian cancer [178], breast cancer [179], and glioblastoma [180, 181]. And HSP60 knockdown inhibits tumor growth in xenograft models of pancreatic cancer [173] and glioblastoma [181]. HSP60 knockdown leads to severe deficiencies in mitochondrial functions, which hinder cell growth and survival. For example, in glioblastoma, reduction in HSP60 increases ROS, which in turn activates the AMPK pathway to inhibit protein translation and slow cell proliferation [180]. In ovarian cancer, knockdown of HSP60 suppresses pathways related to OXPHOS and alters metabolic pathways to allow accumulation of adenine, which activates the adenine-AMPK pathway, suppresses the mammalian target of rapamycin (mTOR) pathway, and inhibits cancer progression [178]. In pancreatic cancer, HSP60 knockdown reduces the expression of subunits in OXPHOS Complexes I, III, IV, and V, disrupts the formation of Complexes I and III, and blocks mitochondrial respiration and ATP production [173]. Consequently, diminished ATP deters the phosphorylation of Erk1/2 and promotes apoptotic activity [173]. As shown in various cancer types, HSP60 associates with cyclophilin D to inhibit cyclophilin D-mediated mitochondrial permeability transition [181]. As such, HSP60 knockdown triggers mitochondrial permeability transition, cytochrome c release, and loss of mitochondrial membrane potential [181]. In addition to direct mitochondrial functions, HSP60 regulates the expression and release of IL-8 in prostate and colon cancers, possibly via transforming growth factor-beta (TGF-*β*), to enhance cell survival [182].

Overexpression of HSP60 enhances the migratory and invasive abilities of FADU cells and promotes the development of metastatic nodules in the lung [183]. The increased *β*-catenin levels and transcriptional activity due to HSP60 overexpression are believed to underlie this metastatic phenotype. HSP60 directly interacts with *β*-catenin [183]. The transcription factor c‐MYC induces overexpression of HSP60, which causes the transformation of Rat1a cells [184].

HSP60 interacts with various cellular proteins to exert biological functions. HSP60 complexes inhibit clusterin to promote cell survival in neuroblastoma [185]. HSP60 can also form a complex with cell cycle and apoptosis regulator protein 2 (CCAR2) and bind the anti-apoptotic protein survivin to promote cell survival [177, 186]. HSP60 is involved in hepatocyte growth factor (HGF)-induced ERK activation to promote cell migration in hepatocellular carcinoma [187].

Although HSP60 is best known as a mitochondrial chaperone and is predominantly located in the mitochondria in non-malignant cells, non-chaperone activity of HSP60 has been reported to be present in the cytoplasm, plasma membrane, and extracellular space of cancer cells [188–190]. HSP60 accumulates in the cytoplasm during apoptosis [188]. Depending on the apoptotic stimuli, this can occur with or without mitochondrial release and HSP60 can display either pro-apoptotic or pro-survival properties, respectively [188]. As a pro-apoptotic protein, HSP60 localized in the cytoplasm assists in the cleavage and activation of procaspase 3 [188, 191]. As a pro-survival protein, cytoplasmic HSP60 is known to bind and restrain p53 to inhibit p53-dependent upregulation of pro-apoptotic Bax [192]. Additionally, cytoplasmic HSP60 can directly bind Bax/Bak to block their translocation to the mitochondria, and thus, interferes with apoptosis [193, 194]. Furthermore, cytoplasmic HSP60 plays a role in TNF-induced expression of SOD1 and Bfl-1/A1, and activation of IKK/NF-κB [195].

Various studies have observed HSP60 on the surface of cancer cells [190, 196–198]. HSP60 proteins localized in the cell membrane are suggested to play a role in the metastatic process [198]. For example, HSP60 can activate* α*3*β*1 integrin [199], a transmembrane receptor that promotes adhesion of breast cancer cells to metastatic sites [200, 201]. HSP60 at the cell surface can also be actively exported into the extracellular space by exosomes. Exosomes are nanometer-sized membrane vesicles containing protein, lipids, and DNA, which are released into the circulation and then taken up by cells in neighboring or distant areas as a means of cell–cell communication [202, 203]. A study by Campanella et al. demonstrated that HSP60 is present on the cell membrane and exosomal membrane and in the Golgi apparatus of cancer cells [189]. They propose a process by which HSP60 present at the cell membrane is internalized by lipid rafts and packaged into multivesicular bodies (MVB) for secretion via exosomes. The Golgi apparatus can assist in transferring cytoplasmic HSP60 into MVB or releasing them from the cell as free, soluble HSP60 [189]. HSP60 can be modified by glycosylation in the endoplasmic reticulum before release, presumably affecting the immunological properties of HSP60 [204]. Overall, exosomal release allows proximate and distant circulation of HSP60.

Interestingly, HSP60 is not overexpressed in all cancers and does not necessarily associate with a poor prognosis in patients. For example, HSP60 is downregulated in bronchial cancer [205], colorectal cancer [206], clear cell renal cell carcinoma [207], and hepatocellular carcinoma [208]. Interestingly, patients with esophageal squamous cell carcinoma [209], clear cell renal cell carcinoma [210], and hepatocellular carcinoma [208] experience better survival rates when their tumors display elevated expression of HSP60. Furthermore, overexpression of HSP60 suppresses cell proliferation in clear cell renal cell carcinoma [207] and inhibits invasive activity in hepatocellular carcinoma [208]. This highlights the multimodal functions of HSP60 across various cancers.

### Heat shock protein 10 (HSP10)

Compared to HSP60, less is known about the HSP60 binding partner HSP10. HSP10 is overexpressed in astrocytoma [211], oral squamous cell carcinoma [212], nasopharyngeal carcinoma [213], large bowel carcinoma [169, 174]. HSP10 is upregulated early during prostate tumorigenesis [168], and levels of HSP10 have been shown to increase throughout the progression of large bowel carcinoma [169]. HSP10 correlates with pathological grade in oral squamous cell carcinoma [212] and nasopharyngeal carcinoma [213], lymph node metastasis in oral squamous cell carcinoma [212], nasopharyngeal carcinoma [213], and large bowel carcinoma [174], and recurrence in astrocytoma [211]. High HSP10 is associated with reduced overall survival in astrocytoma [211], oral squamous cell carcinoma [212], and nasopharyngeal carcinoma [213].

### Mitochondrial heat shock protein 70 (mtHSP70)

Mitochondrial heat shock protein 70 (mtHSP70) is a member of the HSP70 chaperone family and is predominantly located in the mitochondria [214]. It contains a nucleotide-binding domain and a substrate-binding domain [215]. Similar to HSP60, mtHSP70 holds a role in housekeeping and mediates the refolding of unfolded proteins [216]. Multiple studies highlight the protective effect of mtHSP70 on cancer cells and thereby increasing malignancy in cancer.

The chaperone mtHSP70 is upregulated in melanoma [217] as well as cancers of the liver, kidney, thyroid, breast, brain, ovary, lung, and colon [218–223]. Expression levels of mtHSP70 correlate with various clinical features. For example, increased expression of mtHSP70 in breast cancer indicates higher histological grade and decreased survival [224]. Furthermore, mtHSP70 is increased in invasive ductal carcinoma relative to ductal carcinoma in situ and correlates with lymph node metastasis [224]. Similarly, in both hepatocellular carcinoma and non-small cell lung cancer, elevated levels of mtHSP70 are associated with advanced tumor stage, metastasis, and reduced overall survival [220, 223, 225].

Knockdown of mtHSP70 inhibits proliferation, migration, and invasion of various cancer lines [218, 222, 225]. Notably, studies in neuroblastoma indicate that reduction in mtHSP70 is associated with Drp-1-dependent mitochondrial fragmentation [226]. Furthermore, mtHSP70 knockdown leads to diminished levels of p‐ERK1/2, p‐c‐Raf [222], the receptor tyrosine kinase RET (rearranged during transfection), and anti-apoptotic proteins, Bcl-2, Bcl-xL, and Mcl-1 [218].

Overexpression of mtHSP70 induces malignant transformation of fibroblasts, enhancing proliferation and tumor formation in mice [227]. In breast cancer, augmenting mtHSP70 increases the levels of stemness markers such as ATP-binding cassette transporter G2 protein (ABCG2), octamer-binding transcription factor 4 (OCT-4), CD133 and enhances resistance to chemotherapy [228]. In addition, overexpression of mtHSP70 supports EMT transition and metastatic activity in breast cancer.

Elevated mtHSP70 downregulates epithelial markers, upregulates mesenchymal markers, and increases the migratory and invasive abilities of breast cancer cells [221, 228, 229].

Mitochondrial HSP70 can bind various proteins to produce an anti-apoptotic response. For example, mtHSP70 interacts with p53 in response to stressors such as cisplatin [230]. During stress, mtHSP70 binds and prevents nuclear accumulation of tumor suppressor p53, thereby suppressing the transactivation of pro-apoptotic gene targets [231–233]. In response to mtHSP70 knockdown, hepatocellular carcinoma cells undergo p53-mediated apoptosis [230]. On the other hand, in HepG2, a cell line that lacks mtHSP70-p53 interactions, interference with mtHSP70 expression has no effect on cell viability [230].

ERK phosphorylates HIF-1*α* to increase nuclear translocation and promote its transcriptional activity [234]. During hypoxia, if ERK is inactive and unable to phosphorylate HIF-1*α*, mtHSP70 binds and localizes HIF-1*α* to the outer membrane of the mitochondria [235]. HIF-1*α* then associates with voltage-dependent anion-selective channel 1 (VDAC1) and hexokinase-II (HK-II) to prevent apoptosis and promote survival. This mechanism has been shown to protect HeLa cells from etoposide- and doxorubicin-induced death [235].

Interactions between mtHSP70 and other protein targets have also been reported in cells with *BRAF* mutations. In this setting, deregulated MEK-ERK signaling increases interactions between adenine nucleotide translocase 3 (ANT3) and peptidyl-prolyl isomerase cyclophilin D, greatly increasing mitochondrial permeability and promoting mitochondria-mediated cell death [236]. However, mtHSP70 directly interacts with ANT3 to inhibit ANT3–cyclophilin D interactions and secure cell survival [236]. In addition, mtHSP70 can help overcome v-raf murine sarcoma viral oncogene homolog (BRAF) mutation-induced growth arrest signaling by inhibiting Raf-induced MEK/ERK activity through inhibition of MEK1/2 protein expression and ERK1/2 phosphorylation [217].

Interestingly, mtHSP70 has also been reported to form complexes with HSP60 [237], although the function of this complex remains to be fully elucidated. However, it is possible that this study has detected a transient interaction in which HSP60 is mediating the folding of mtHSP70 that has been recently imported into the mitochondria.

### Lon peptidase (LONP1)

As mitochondrial proteins undergo folding, they are prone to aggregation. This is especially true in the presence of oxidative stress [238–240]. LONP1, which is composed of a hexagonal cylinder with a large, unfolding, and degradation chamber [241], acts as a protease that cleaves aggregates into short peptides that are cleared from the mitochondria. In doing so, LONP1 maintains mitochondrial proteostasis [142]. LONP1 does not appear to degrade folding intermediates of mitochondrial matrix proteins, indicating that mitochondria preferentially utilize chaperones to prevent aggregates and restore protein functions. Proteolytic actions are taken only as a final measure.

LONP1 is upregulated in melanoma [242], prostate cancer [243], pancreatic cancer [244], and colorectal cancer [242]. Notably, LONP1 increases during colorectal cancer progression and is particularly increased in colorectal samples with mutated p53 or *β*-catenin [245]. Additionally, hypoxia increases LONP1 expression in PCa [243]. High expression of LONP1 correlates with reduced overall survival in neuroblastoma, breast cancer, colorectal cancer, renal cell carcinoma [243], and metastatic cohorts of melanoma [242].

Knockdown of LONP1 leads to reduced proliferation in melanoma [242], colorectal cancer [242], pancreatic cancer [244], and PCa cells [243]. In vivo, LONP1 knockdown inhibits the growth of prostate [243] and colorectal tumors [242] and inhibits the formation of metastatic lesions from primary prostate [243] and melanoma tumors [242]. A deficiency in LONP1 expression in mice inhibits the formation and growth of azoxymethane- and dextran sulfate-induced colorectal tumors [242]. Similarly, these animals are also resistant to 12-dimethylbenzanthracene and tetradecanoylphorbol acetate (DMBA/TPA)-induced skin papilloma [242]. In addition, several have implicated LONP1 in gastric carcinogenesis. In response to *H. pylori* infection, LONP1 expression increases in gastric cancer cells [246], and LONP1 is necessary for *H. pylori*-induced gastric cell proliferation and promotes *H. pylori*-induced metabolic switch to glycolysis. [246].

The pro-tumor effects of LONP1 can be partially attributed to LONP1-mediated regulation of Bcl-2 in melanoma [242], cyclin D1 in pancreatic cancer [244], and *β*-catenin in colorectal cancer [245]. LONP1 also appears to have a role in EMT. As shown in pancreatic cancer cells, LONP1 knockdown increases the expression of the epithelial marker claudin-1 and decreases the mesenchymal marker vimentin as well as transcription factors snail and slug [244]. In addition, decreases in matrix metalloproteinase (MMP2), MMP9, and p-JNK are observed after LONP1 knockdown. Overall, these features are believed to contribute to a resulting decrease in migratory and invasive abilities of LONP1 knockdown cells [244].

Alterations in LONP1 expression lead to various dysfunctions of the mitochondria. In PCa, knockdown of LONP1 associates with an accumulation of misfolded subunits of OXPHOS Complex II and V, reduced assembly of OXPHOS Complexes I, III, IV, and V, as well as inhibition of activities of OXPHOS Complexes I, II, and V [243]. Consequently, mitochondrial respiration and ATP production are decreased. [243]. Studies in gastric cancer demonstrate that LONP1 knockdown reduces mitochondrial respiration [246]. In melanoma, LONP1 knockdown inhibits the formation of OXPHOS Complexes I and III and reduces the activities of OXPHOS Complexes I, II, and III [242]. Consequently, cells experience an increase in mitochondrial fragmentation and ROS levels as well as a decrease in mitochondrial respiration and ATP.

Interestingly, overexpression of LONP1 also leads to deleterious effects. LONP1 overexpression in cancer cells lowers the activities of Complexes I, II, III, and IV in conjunction with reduced mitochondrial respiration [242]. A study in squamous cell carcinoma further demonstrates that while LONP1 overexpression induces mtROS production through Complex I, mtROS can activate Ras and MAPK signaling and promote the survival of these cells [247]. Additionally, LONP1 binds and stabilizes Hsp60–mtHsp70 complexes, particularly under oxidative stress conditions [248]. This interaction is proposed to inhibit apoptosis. However, knockdown of LONP1 has no effect on the individual expression levels of HSP60 and mtHSP70 [248].

### Caseinolytic protease (ClpP)

CIpP forms a tetradecameric cylinder, similar to LONP1, which accepts protein substrates for degradation [249]. Notably, various adaptors can bind to ClpP to influence substrate selectivity [250]. In addition, accessory proteins deliver protein substrates to ClpP for degradation.

ClpP is upregulated in breast cancer, PCa, and acute myeloid leukemia [251–253]. High ClpP expression correlates with poor recurrence-free survival in breast cancer [251]. Elevated ClpP levels promote cisplatin resistance in cervical cancer cells by inhibiting the accumulation of cisplatin in these cells, and partly through increasing the expression of copper efflux pump ATP7A [254].

Knockdown of ClpP is associated with reduced proliferation, migration, and invasion of various cancer cells [251–253]. Xenograft studies demonstrate that ClpP knockdown suppresses the growth of PCa-derived liver metastases [252]. These characteristics are likely due to the observed reductions in the expression levels of cyclin A, cyclin B1, cyclin D1, and MMP7 [251, 252], as well as the inhibition of PI3K and AKT activation after ClpP knockdown [251]. Conversely, overexpression of ClpP increases migratory and invasive activity in breast cancer [251]. Interference with ClpP expression perturbs mitochondrial function, as evidenced by an increase in mtROS, accumulation of misfolded mitochondrial Complex II subunits, hyperoxidation of mitochondrial peroxiredoxin III, reduced Complex II activity but increased Complex V activity, and overall diminished mitochondrial respiration [252, 253].

## Concluding remarks

Although mitochondria contribute to uncontrolled proliferation throughout the various stages of cancer [3], mitochondria are also highly vulnerable in cancer cells. Tumors generate increased levels of oxidative stress and proteotoxic stress that leave mitochondria in a fragile, dysfunctional state [4–6]. Overall, the literature supports the idea that UPR^mt^, a mitochondrial stress response observed in *C. elegans* [10], serves as an important support system in cancer to maintain mitochondrial health and promote tumor growth (Figs. 1, 2, 3).

Multiple questions regarding the regulation of UPR^mt^ in the cancer setting remain. Specifically, the mechanism by which ATF5 regulates UPR^mt^ in cancer must be further addressed. Under non-stress conditions in the *C. elegans* model, ATFS-1, the homolog of ATF5, accumulates in the mitochondria and is degraded by LONP1 [113]. When mitochondrial stress is present, mitochondrial import of ATFS-1 into the mitochondria is reduced. Instead, ATFS-1 accumulates in the nucleus where it can function as a transcription factor and upregulate components of UPR^mt^ to alleviate mitochondrial stress. It is unknown if ATF5 similarly translocates from mitochondria to the nucleus in mammals. In addition, no study has investigated if ATF5 has a direct transcriptional role in the mitochondria, where it could bind mtDNA and directly regulate the expression of mitochondrial-encoded proteins.

Furthermore, it is important to recognize that ATF5 is likely not the sole transcription factor responsible for the activation of UPR^mt^ in cancer. Although ATF5 appears to be the only mitochondria-specific mediator of UPR^mt^, it remains likely that ATF5 functions in parallel with other key transcription factors that contribute to both forms of UPR in the mitochondria and endoplasmic reticulum. This suggests cross talk between UPR^mt^ and UPR^er^. The extent to which established transcription factors of UPR^er^ can induce UPR^mt^ relative to the mitochondria-specific ATF5 is unknown. As we continue to understand the specific roles of ATF5 and other members of UPR^mt^ in cancer, we become better equipped to develop pharmacological agents that can target UPR^mt^ as a novel form of cancer therapy.

Current findings clearly suggest that cancer cells are highly reliant on the UPR^mt^ for growth and progression, and therefore, aggressive and resistant cancers such as PCa possess robust activation of UPR^mt^ [255, 256], which could be targeted for developing novel therapies to cure cancer. Recently, we have identified an inhibitor of UPR^mt^, a promising new anticancer agent for cancer such as PCa. This unique UPR^mt^ inhibitor does not rely on androgen receptor-mediated signaling and, thus, will establish a foundation for the development of novel therapies to cure resistant PCa irrespective of AR status. Therefore, targeting this longevity promoting function of mitochondria, the UPR^mt^, will be an attractive and feasible target for aggressive and metastatic cancers.

## Data Availability

Not applicable.

## Abbreviations

UPR^mt^: Mitochondrial unfolded protein response
mtROS: Mitochondrial reactive oxygen species
RAS: Rat sarcoma virus
MYC: Myelocytomatosis
EGFR: Epidermal growth factor receptor
PTEN: Phosphatase and tensin homolog (PTEN)
MAPK: Mitogen-activated protein kinase
AKT: Ak strain transforming
AMPK: AMP-activated protein kinase
ERK: Extracellular signal-regulated kinase
NF-κB: Nuclear factor kappa B
TMEM126A: Transmembrane protein 126A
BCL-2: B cell lymphoma 2
MCL-1: Myeloid leukemia 1
Bcl-xL: B cell lymphoma-extra
mTOR: Mammalian target of rapamycin
MMP: Matrix metalloproteinase
ROS: Reactive oxygen species
OXPHOS: Oxidative phosphorylation
ETC: Electron transport chain
mtDNA: Mitochondrial DNA
TCA: Tricarboxylic acid
PCa: Prostate cancer
ND5: NADH dehydrogenase subunit 5 gene
PIP3: Phosphatidylinositol 3,4,5-trisphosphate
CDK1: Cyclin-dependent kinase 1
PKD1: Protein kinase D1
HMGB1: High-mobility group box 1
RAGE: Receptor for advanced glycation end products
TME: Tumor microenvironment
HIF-1*α*: Hypoxia-inducible factor-1*α*
PHDs: Prolyl hydroxylases
NSCLC: Non-small cell lung cancer
EMT: Epithelial–mesenchymal transition
SOD: Superoxide dismutase
HSP60: Heat shock protein 60
HSP10: Heat shock protein 10
mtHSP70: Mitochondrial heat shock protein 70
ClpP: *Caseinolytic protease*
*MTS*: Mitochondrial targeting sequence
NLS: Nuclear localization sequence
LONP1: Lon peptidase 1
ATF5: Activating transcription factor 5
ER: Endoplasmic reticulum
CHOP: C/EBP homologous protein
ER*α*: Estrogen receptor alpha
SIRT3: Sirtuin deacetylase 3
HSF1: Heat shock factor-1
UPR^er^: Endoplasmic reticulum unfolded protein response
NES: Nuclear export sequence
CCAR2: Cell cycle and apoptosis regulator protein 2
HGF: Hepatocyte growth factor
MVB: Multivesicular bodies
